# Community-based care of children affected by AIDS in Swaziland: a gender-aware analysis

**DOI:** 10.1017/S1463423618000774

**Published:** 2018-11-16

**Authors:** Michelle R. Brear, Pinky N. Shabangu, Karin Hammarberg, Jane Fisher, Helen Keleher

**Affiliations:** 1 Adjunct Research Fellow, Monash University, School of Public Health and Preventive Medicine, Jean Hailes Research Unit, Melbourne, Victoria, Australia; 2 Postdoctoral Research Fellow, Afromontane Research Unit, University of the Free State–Qwaqwa, South Africa; 3 Community-based researcher, Manzini, Swaziland; 4 Monash University, School of Public Health and Preventive Medicine, Jean Hailes Research Unit

**Keywords:** children affected by AIDS, community-based care, gender discrimination, participation, poverty, social power

## Abstract

**Background:**

Care of children affected by AIDS in Swaziland is predominately provided by families, with support from ‘community-based responses’. This approach is consistent with United Nations International Children’s Fund’s (UNICEF) framework for the protection, care and support of children affected by AIDS. However, the framework relies heavily on voluntary caregiving which is highly gendered. It pays limited attention to caregivers’ well-being or sustainable community development which enables more effective caregiving. As a result, the framework is incompatible with the social justice principles of primary health care, and the sustainable development goals (SDGs).

**Aim:**

Our aim was to examine the effects and gender dimensions of providing voluntary, community-based, care-related labour for children affected by AIDS.

**Methods:**

We conducted multiple-methods research involving an ethnography and participatory health research, in a rural Swazi community. We analysed data related to community-based responses using an abductive, mixed-methods technique, informed by the capabilities approach to human development and a gender analysis framework.

**Findings:**

Two community-based responses, ‘neighbourhood care points’ (facilities that provide children meals) and the ‘*lihlombe lekukhalela*’ (child protector) program were being implemented. The unpaid women workers at neighbourhood care points reported working in challenging conditions (eg, lacking labour-saving technologies), insufficient and diminishing material support (eg, no food), and receiving limited support from the broader community. Child protectors indicated their effectiveness was limited by lack of social power, relative to the perpetrators of child abuse. The results indicate that support for community-based responses will be enhanced by acknowledging and addressing the highly gendered nature of care-related labour and social power, and that increasing access to material resources including food, caregiver stipends and labour-saving technologies, is essential. These strategies will simultaneously contribute to the social and economic development of communities central to primary health care, and achieving the poverty, hunger, gender and work-related SDGs.

## Introduction

### Care of children affected by AIDS in Swaziland

Providing care to children affected by AIDS is a major challenge in the Kingdom of Swaziland, an agrarian nation of 1.2 million people in southern Africa (UNICEF Swaziland, 2012). Swaziland has the highest prevalence of HIV globally (UNAIDS, 2014). Children under 18 years comprise 45 per cent of the Swazi population and up to 20 per cent of all Swazi children have experienced the death of at least one parent (UNICEF, 2013). The majority of children whose parents have died are cared for in rural communities, by members of their extended families. Women perform almost all child care-related labour (Braithwaite *et al*., 2013).

The development of a National Plan of Action for Orphans and Vulnerable Children (Government of the Kingdom of Swaziland, 2006) marked the beginning of a Swazi Government–UNICEF partnership to strengthen care for children affected by AIDS. The plan identified several community-based responses, being implemented in two rural Swazi communities, which could be ‘scaled-up’ to other communities. Amongst these, neighbourhood care points (NCPs) and the *Lihlombe Lekukhalela* (child protector) program, have been the most prominent (UNICEF Swaziland, 2012).

#### Neighbourhood care points

NCPs were intended to be facilities that would provide a full spectrum of child health and development services. It was envisaged that NCPs would operate by ‘mobilizing communities’ (UNICEF Swaziland, 2006: 9) and ‘be community-driven and owned’ (UNICEF Swaziland, 2006: 6), so that after establishment they would require ‘minimal subsequent servicing’ from external organisations (Regional Hunger and Vulnerability Program [RHVP], 2007: 4). However, providing meals using food supplied by external donors, quickly became, and remains, the primary function of NCPs. In 2012, over 1500 NCPs had been established in Swazi communities. Collectively they fed approximately half of the country’s 100,000 three- to five-year-old children (UNICEF Swaziland, 2012). NCPs are considered the most successful of Swaziland’s community-based responses to support families caring for children affected by AIDS (RHVP, 2007) and highly cost-effective relative to the benefits they provide (Dlamini, 2007).

#### Lihlombe lekukhalela (child protector)

*Lihlombe lekukhalela* (literally ‘shoulder to cry on’) loosely translates to ‘child protector’. It refers to a community volunteer responsible for identifying and supporting abused children, including those subjected to child labour, abuse and neglect. Child protectors are also expected to play a role in sensitizing their communities to child rights (UNICEF Swaziland, 2007). The *Lihlombe Lekukhalela* project was initiated by UNICEF in response to reports of abuse of Swazi children, including beating, overwork and sexual harassment (UNAIDS, 2006). By 2007, over 8000 child protectors had been trained, for two to three days, in child rights, counselling and identifying and reporting cases of abuse to police and community leaders (UNICEF Swaziland, 2007). By 2014, numbers had expanded to 10,000 volunteer child protectors (Ferriera-Meyers, 2014).

Evaluations of the *Lihlombe Lekukhalela* program’s impact, conducted through short-term consultancies, have reported mixed results. They suggest child protectors are known to children in communities and have increased awareness of child abuse and rights. However, the child protectors face numerous challenges, including difficulty accessing funding, and working with community members whose beliefs about what constitutes child abuse diverge from international definitions (eg, thinking that giving children rights makes them undisciplined) (UNICEF Swaziland, 2007; Ferriera-Meyers, 2014).

### The community-based care framework for children affected by AIDS

The approach adopted by Swaziland, of relying on individual families and entire communities to support the needs of children affected by AIDS, is referred to as ‘community-based care’. Advocated by UNICEF (UNICEF, 2007), the model has been adopted in national policies throughout sub-Saharan Africa. It is considered a sustainable approach to caring for children affected by AIDS because (compared to institutional care) it is cost-effective (Desmond *et al*., 2010), participatory and community based (Foster *et al*., 2010).

The UNICEF framework’s strategy of ‘supporting community-based responses’ (UNICEF, 2007: 12) by providing material, technical or other assistance to community-led interventions, is fundamentally a primary health care approach. Social and economic development, community participation and providing community-level primary health care services were core Alma Ata strategies for achieving health for all (WHO, 1978). However, the Alma Ata Declaration goal of ‘health for all’, recently reiterated in the sustainable development goals (SDGs) remains elusive in communities affected by AIDS. The need to strengthen the care of children affected by AIDS, who are vulnerable because they have a high risk of food insecurity, poverty and abuse, is widely recognised (Chandan and Richter, 2009). Gender analysis of community-based care policy and practice has a potentially important role to play in strengthening children’s care, by protecting and promoting the rights of caregivers (Steege *et al*., 2018).

### Gender analysis of community-based care

Gender analysis seeks to, ‘understand gender power relations and norms and their implications’ (Morgan *et al*., 2016: 1070), by considering what women and men do, have and can decide. It is pertinent to protecting and promoting the rights of unpaid caregivers, internationally (Steege *et al*., 2018) and in the southern African region (Daniels *et al*., 2012), because the division of care-related labour is highly gendered. For example, in Swaziland, 92 per cent of child protectors are women (Ferriera-Meyers, 2014) and most NCPs are run by female volunteers (UNICEF Swaziland, 2012: 31). This highly gendered division of labour is detrimental. It exacerbates women’s economic marginalisation because it constrains their participation in secure, paid employment (Antonopoulos and Hirway, 2010). In many cases, women are only available to provide care, because they cannot find decent jobs (Daniels *et al*., 2012).

However, in low- and middle-income countries (LMICs), the community health workforce is often still analysed and presented in gender-neutral terms (Morgan *et al*., 2018). This true of Swazi (Government of the Kingdom of Swaziland, 2006) community-based care policies, which typically use gender-neutral terms like ‘families’ and ‘communities’, to refer to the women providing care to children affected by AIDS. For example, one report refers to ‘mobiliz[ing] whole communities’ (UNAIDS, 2006: 7), and children being ‘cared for by family relatives’ (UNAIDS, 2006: 11). Failure to acknowledge the gendered division of care-related labour, masks the effects of gendered power, and potentially the exploitation of women (Morgan *et al*., 2016). It situates and socialises children to perceive, women caring for children without remuneration or reward as ‘natural’ (Nussbaum, 2000).

In Swaziland, the potential for the NCP model to exploit women’s unpaid work and the need to protect and promote the rights of NCP caregivers were considered (albeit briefly) in 2008, when UNICEF secured stipends for NCP caregivers of 200 Swazi emalangeni (~USD20) per month. However, the strategy was opposed by government representatives and UNICEF, and the stipends were rapidly withdrawn at the request of the international funder, after staff of the non-governmental organisations charged with dispersing the stipends reported they reduced ‘community ownership’ and dangerously increased dependency on donors (UNICEF Swaziland, 2012). Now, NCP caregivers sometimes receive food parcels as recognition for their work. A recent report concluded that now ‘it is impossible to find anyone who was in favour of the stipends except, perhaps, those who hoped to receive them’ (UNICEF Swaziland, 2012: 31). Notably, ‘those who hoped to receive’ stipends, were the women caring for children affected by AIDS, whose voices were largely excluded from the research on which the report (UNICEF Swaziland, 2012) was based.

The voices of women who provide care are often excluded from, or assumed to be represented by non-governmental organisations, in research about community-based care (Daniels *et al*., 2012). However, listening directly to the women who provide care is pertinent to gender analysis (Morgan *et al*., 2016) and improving community-based care (Daniels *et al*., 2012). Policies and programs cannot be optimally effective, if they do not consider caregivers firsthand knowledge of the strengths, limitations and challenges of community-based care (Daniels *et al*., 2012).

The aim of this study was to conduct a gender analysis of volunteer caregiving in community-based responses for children affected by AIDS in Swaziland. We particularly wanted to understand the gendered power dimensions of caregiving from the perspectives of women who provided community-based care. We also wanted to investigate how gendered power impacted on the care of children affected by AIDS.

## Methods

We conducted multiple-methods research about community-based responses for children affected by AIDS in a rural Swazi community implementing NCP and *lihlombe lekukhalela* interventions. This article reports the integrated findings of data generated through the two components of the study, an ethnography of daily life at a NCP and preschool involving participant observation, and participatory health research (PHR) about a community caring for children affected by AIDS, involving a census survey and focus group discussion.

### Setting

The first author (MB) selected the community purposively because she had been participating in the implementation of community-based responses for children, for over five years. Specifically, she had participated in establishing and operating the community preschool and the NCP to which it was attached (one of three NCPs in the community). Through these historic interactions, members of the community and MB had established a degree of trust and mutual interest that is considered essential for successful, equitable PHR (Israel *et al*., 2013).

### Recruitment

The study was approved by the Monash University Human Research Ethics Committee (CF12/2637–2012001422 and CF13/994–2013000486) and the Swaziland Scientific and Ethics Committee (MH599C). Community-level consent was provided in writing by the *umphakatsi* (the traditional local governance authority). Community members provided oral informed consent and community co-researchers provided written informed consent for MB to conduct participant observation of daily life at one of three NCP in the community, and of the PHR process. Census respondents provided verbal consent before completing the survey by face-to-face interview. Potential focus group respondents were selected by stratified random sample from amongst all members of households in which the census respondent provided consent for members to be invited. They were invited by community co-researchers who visited their household and gave (or if necessary read to) them an explanatory statement. Those aged over 17 who agreed to participate provided written informed consent. Those under 18 years of age were invited to participate after a guardian provided written informed consent; they gave oral consent. All participants were given a token of appreciation for their participation of USD1 or a gift of equivalent value.

### Data collection

The ethnography involved structured and unstructured observations of participation in care-related labour at the NCP and preschool, to elicit information about the everyday reality and gender dynamics of community-based caregiving. Structured observations about the number of visitors, the visitors’ sex and the purpose of the visit were documented daily by two community co-researchers who were also preschool teachers. MB collected unstructured observations while participating in the daily activities of the NCP and working in partnership with community co-researchers to design the PHR methods and tools. All ethnographic data were collected in English.

Community co-researchers participated in all aspects (as defined by Brear *et al*., 2018a) of the mixed-methods PHR. It involved: (1) a paper-based demographic and health census survey intended to determine the population size and elicit information about the spread of health-related characteristics, including use of community-based care and (2) focus group discussions (FGDs) with (a) male and female adult and youth community members, stratified by age and sex, and (b) groups of key informants, including NCP caregivers and community health workers (CHW) (of which *Lihlombe Lekukhalela* were one type). Focus group discussions were employed to elicit community members’ and caregivers’ perspectives about the benefits and problems people faced living and/or volunteer caregiving in the community. All PHR data were collected in siSwati (the participants’ mother tongue).

### Data analysis

Quantitative data were entered to a database and analysed using descriptive statistics. Hand-written participant observation notes were transcribed by MB within 24 hours of collection. Focus group discussions were audio recorded in siSwati (the participants’ mother tongue); each was translated twice, including once by a community co-researcher (including the second author, PS), directly into English, and transcribed. The two English transcripts were checked for discrepancies and amended accordingly by two bilingual co-researchers (including PS). Pseudonyms chosen by PS were allocated to each FGD participant, and used to reference in-text quotations, according to a standardised system (Table 1).

**Table 1:** Data sources and style for in-text references

Data	In-text citation structure	In text citation example
Unstructured participant observation	PODD/MM/YYYY	PO13/04/2013
FGD – community member	Pseudonym-Sex (M/F)-AGEyo	Vusi-M-45yo
FGD – community health worker	Pseudonym-CHW Role^a^ – Age group^b^	Gugu-NCP Caregiver-30s

a Roles are either: neighbourhood care points (NCP) Caregiver; Child Protector or community health workers (CHW) (used to refer to all other CHWs). Sex is not recorded and all pseudonyms used are female as all but one of the CHWs were female.

b Age groups are 20–29; 30–39, 40–49, 50–59, over 60.

Focus group discussion data related to community-based responses for children affected by AIDS were identified in MB’s primary analysis. These data were analysed by MB using an abductive mixed-methods approach, that is incorporating inductive (informed by the data) and deductive (informed by theory) perspectives, and integrating results from data generated using different methods, in ways that highlighted explanatory, confirmatory and/or contradictory findings (Onwuegbuzie and Combs, 2010). Specifically, the focus group discussions and unstructured participant observation data were analysed thematically. The results of univariate statistical analysis of quantitative (structured participant observation and census) data were integrated into the text of the qualitative findings. Feedback on the results presented in this article was obtained from NCP caregivers and community co-researchers in ‘member checking’ (Miles *et al*., 2014) sessions.

The analysis was informed by the capabilities approach to human development. This theory conceptualises care-related labour in relation to its impact on women’s and children’s capabilities (what they can ‘do and be’). It proposes that lack of recognition and valuation of women’s care-related labour is a barrier to human development, not only because it constrains women’s freedom. It also socialises children to accept a gendered division of care-related labour that is far from ‘natural’ and highly discriminatory (Nussbaum, 2000). We applied capability theory, within Morgan *et al*.’s (2016) gender analysis framework, which recommends disaggregating data by sex, to enable consideration of gendered power dynamics related to what men and women are expected to, and/or actually do, have access to, and are able to decide.

## Results

Structured participant observation data were collected on 102 of 125 (82 per cent) consecutive school days in two terms (May 2013–November 2013). Unstructured participant observation data were collected on 76 non-consecutive days over 14 months (November 2012–January 2014). The household survey was completed by a respondent from 151 households in the community (99 per cent response rate). It generated demographic data for 1031 household members, including members who lived away from the household most of the time (Table 2). Of the 55 male and 67 female household members invited to participate in focus groups, 29 males (53 per cent) and 55 females (82 per cent) actually participated in 1 of 12 discussions with a mean duration of 61 (range 48–79) minutes and a median of seven (range 4–11) participants.

**Table 2:** Summary statistics for community demographic characteristics

	Female *N* (%)	Male *N* (%)	Total^a^ *N*
Household members			1031
All	509 (50)	519 (50)	1028
*Living in the household most of the time*			*693*
<18	188 (47)	210 (53)	398
≥18	192 (65)	103 (35)	295
*Living away from the household most of the time*			*327*
<18	34 (52)	31 (48)	65
≥18	93 (35)	169 (65)	262

a Only cases with data for each variable were included in the analysis.

The analysis identified four themes: poor working conditions; insufficient and diminishing material support; limited support from the broader community and lack of social power. We first describe the community-based care (NCP and *lihlombe lekukhalela*) interventions in the community. We then present the results for each of these themes.

### Community-based care

The community had two stand-alone NCPs and one preschool-based NCP. The two stand-alone NCPs provided a single daily meal to children under five years of age; one was reported to be used by at least one child in 5 per cent and the other by at least one child in 10 per cent of households in the community, in the 12 months prior to the census. The NCP attached to the preschool provided students (five-year-olds) two meals per day and was used by at least one child in 20 percent of households in the year prior to the census. All the NCPs relied on external donors to provide staple foods (maize meal, cooking oil and legumes). All NCPs also relied on volunteer caregivers to cook and serve meals, and all the caregivers were women.

The *Lihlombe lekukhalela* (child protector) program was less prominent in the community, compared to the NCPs. Most of the community co-researchers had never heard of Child Protectors. For example, when discussing different types of CHWs to invite to focus group discussions, most were only aware of one group (*bagcugcuteli* or rural health motivators). One co-researcher, who was herself a CHW, also knew of a group called *banakekeli* (home-based caregivers). She perceived community police to be the volunteer group responsible for managing child abuse (PO03-01-13). It was only when MB specifically enquired about Child Protectors, that the latter co-researcher reported she had recently become aware of this group (PO09-01-13), and the co-researcher group decided to identify child protectors in the survey. All but one of the child protectors in the community were women.

### Challenging working conditions

What the women who volunteered as caregivers had (or lacked), and what they did (or did not) do, made working at NCPs challenging. The caregivers said their work was challenging because they lacked labour-saving technologies and typically worked alone (a different caregiver ran each NCP, each day). Although there were also teachers present at the preschool-based NCP, MB observed that the caregivers spent much of their time working alone, while the teachers interacted with the students. At this NCP, cooking the food required substantial work, leaving caregivers with little time to interact with children, except to hand them their plates of food.

**Figure 1:**
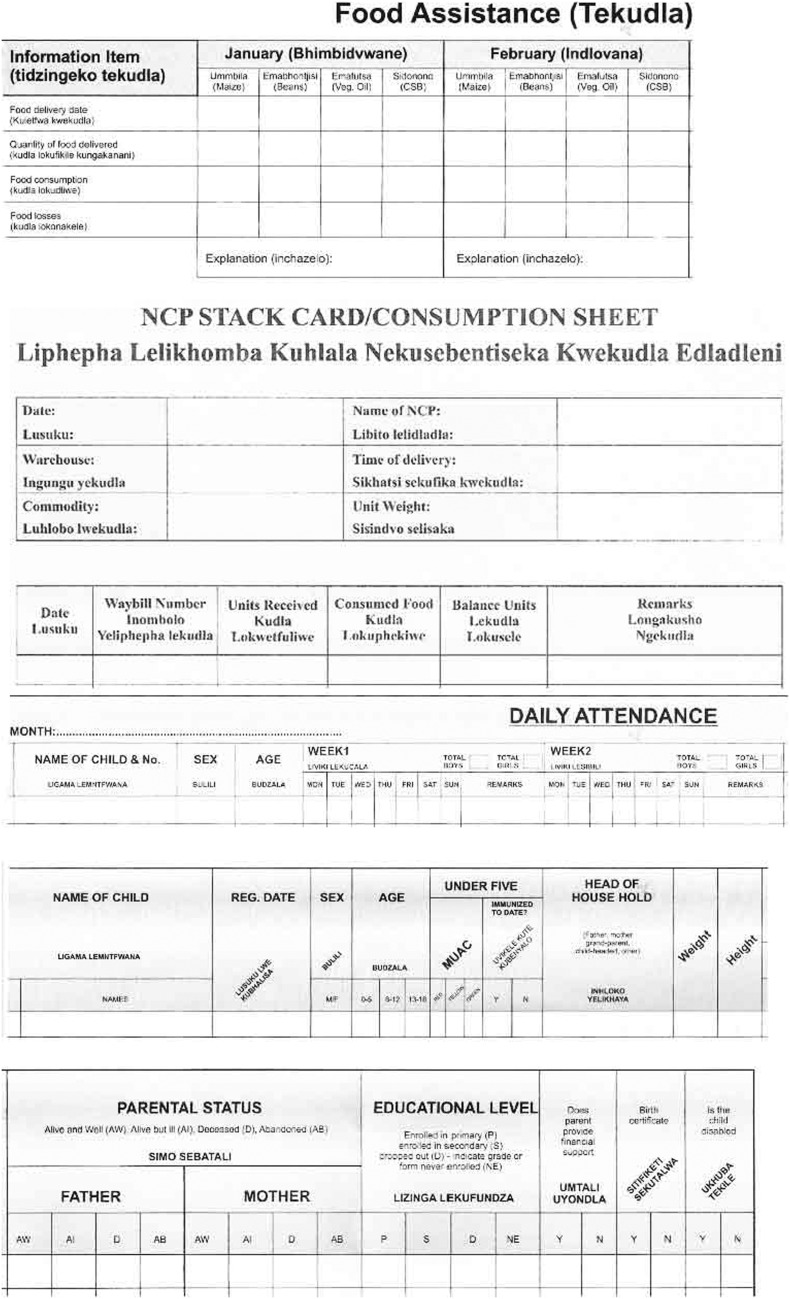
Sections of the file caregivers were required to record information in

An average day for an NCP caregiver involved cooking over an open wood fire, using heavy cast iron pots. The NCP caregivers would also ‘fetch [three 20L buckets of] water [from the river], collect firewood, do some sweeping and cleaning…then cook the food for the children’ (Philile-NCP Caregiver-60s). Other work included washing pots and plates, ‘help[ing] them [children] pray and wash their hands before eating’ (Sphelele-NCP Caregiver-20s), cutting grass so that children ‘cannot be bitten by snakes’ (Gugu-NCP Caregiver-30s) and completing ‘the file’ (Figure 1) for which they needed to bring their own pens (or borrow their children’s school pens). One said:

[The file] stresses us big time… some of the things are written in English and… we have not been in school for a very long time now… you will have to… estimate and deduct so that gives you a problem and sometimes… [the food] does not balance. (Sikhulile-NCP Caregiver-30s)

The preschool NCP caregivers relied on the teachers to complete the file because they could not read and write.

Unsound facilities made working conditions even more challenging. One of the stand-alone NCPs operated from a traditional mud hut and had no toilet. Further:

Grass [from the roof] was blown away because it was windy…so they [NCP caregivers at that facility] are trying to fix it…on the floor they use cow dung. …There is no such thing that they have to clean [the floor] using water because there is no cement. (Philile-NCP Caregiver-60s)

The caregivers at this NCP said the food provided by external donors got eaten by mice because they had nowhere to store it safely and got destroyed when it rained because the roof leaked. Ultimately the food ran out, forcing the NCP to close.

### Insufficient and diminishing material support

Not having food to cook and serve to children, was one aspect of a broader problem with insufficient and diminishing material assistance that the NCP caregivers reported. Periodic closure due to food running out, when external donors did not deliver on time, was considered a problem at all NCPs. The caregivers expected maize meal, cooking oil and legumes to be delivered by external donors on a bi-monthly basis. However, they said that, ‘when the food has finished, it takes a long time for it to be delivered again’ (Sphelele-NCP Caregiver-20s) and as a result, ‘the children will come here and find that there is nothing and they go back home’ (Nomcebo-NCP Caregiver-40s). The preschool NCP did not receive food from external donors between November 2012 (when its construction was completed) and June 2013 (PO25/06/2013).

Sugar and salt were not provided by external donors, nor were ‘different kinds of vegetables like beetroot, spinach, onions, tomatoes so as to balance the nutrients… not to just eat beans [legumes] every day’ (Sikhulile-NCP Caregiver-30s). External donors initially provided all essential operating supplies (including soap, pens, buckets for fetching water and washing dishes, plates for serving children’s food and food scoops for measuring out the correct amount of food to cook each day so that ‘the file’ (Figure 1), in which they recorded food usage and child attendance, would ‘balance’). However, the caregivers reported now having to supply these items themselves for example, ‘bring[ing] the wooden spoons [and] cups for dishing up the relish’ (Nokwanda-NCP Caregiver 26yo) from home and supplying consumables like soap and sugar because, ‘you cannot cook the children the *sidonono* [fortified breakfast porridge] without sugar… [so] we used money from our own pockets [to buy it]’ (Lungile-NCP Caregiver-60s). The caregivers at the preschool NCP relied on the preschool to pay for these additional supplies.

The staple food provided by donors was insufficient to provide more than one meal a day to children and the NCP caregivers were not permitted to give the children raw food to take home and cook. They perceived this created challenges because it meant that home-based caregivers each had to walk to the NCP with the children who were under five year olds. The NCPs were situated up to 3.5 km (round-trip) away from the households that accessed them, and some more distant households reported they did not use NCPs because they were too far away. The home-based caregivers who journeyed to NCPs with children were not entitled to receive a meal.

The caregivers said they expected external organisations to provide the children’s food because they had promised to do so when they started the NCPs. These organisations had also promised to provide food-for-work rations; however, all NCP caregivers agreed that ‘It is of great luck if it happens that we get the food [for-work] once or twice a year’ (Lungile-NCP Caregiver-60s) and that ‘We do not know if it is a must [that the organisations pay us food-for-work] or we are cheated. No one knows’ (Sikhulile-NCP Caregiver-30s). They noted that their ability to grow additional food for the children was constrained because they did not have materials for fencing vegetable gardens, nor irrigation infrastructure. They also said that they would need broader community support to plant, tend and harvest the gardens.

### Limited support from the broader community

Community members were reportedly reluctant to help caregivers with NCP-related work, but quick to gossip about them. For example, the caregivers reported community members saying, “‘leave them alone, they do get paid…” [Yet] we get the food [for-work rations] after a very long time, work and work for nothing leaving our work [at home]’. (Sphelele-NCP Caregiver-20s), and that the caregivers were selling the food at NCPs when ‘trying to raise money for buying the salt and sugar’ by asking parents to send five or ten cents (Zanele-NCP Caregiver-30s).

Ad hoc volunteer work (including fetching water and firewood, gardening, cooking meals and cleaning) performed at the NCPs was also highly gendered. When asked to speak about the work done by men at the NCPs, the caregivers discussed how gender norms in the community meant men did not get involved:

Tenkhosi (age missing): I do not remember getting any support from males in this community. Males? It is only females…

Lungile (65yo): There is no work that men do…

Philile (67yo): They tell themselves that it is women’s work…

Zanele (36yo): You only find women, they know that it is women’s work.

At the preschool NCP, the daily visitor’s survey showed that women (including the four NCP caregivers) collectively made 217 volunteer work contributions during the study period; men did not contribute any volunteer work. However, one man, the father of a preschool student, did make a contribution of volunteer labour the following year (PO11-02-2014).

### Lack of social power

Child protectors indicated the organisation’s effectiveness was limited by child protectors’ lack of social power, saying:

Lihlombe lekukhalela, it is very difficult to work in that organisation. …If we were seriously working, others would go to jail. As a member at Lihlombe lekukhalela you need to have a lawyer because if I can start and tell someone that they are abusing their child, then they take me to their lawyer. …There is a lot you can see but can’t work it properly because [the people abusing children are powerful]… we from this organisation [Lihlombe lekukhalela] need to arrest the police. (Lungelo-Child Protector-50s)

The limited social power Lungelo discusses is also apparent in NCP caregivers accounts of their experiences providing community-based care. For example, they did not have the power to advocate to ensure that they received stipends that they clearly desired (or even to ensure donors provided the food they had been promised). Nor did they have the power to mobilise community members or resources. Indeed they were unable even to mobilise the few cents they needed to buy sugar and salt, and were left with no choice but to take it from their own pockets.

## Discussion

### Strengths and limitations

These results were generated using an innovative, theoretically informed combination of methods and analytical techniques. The study was conducted in a partnership which involved community members in all aspects of the research process. Working with community co-researchers, who were known to the respondents and had no prior experience conducting research, likely influenced responses (potentially in both negative and positive ways). The results are specific to the community in which they were produced. However, poverty, gender discrimination and the model of community-based care for children affected by AIDS (and other primary health care approaches that rely heavily on unpaid community participation) are pervasive in sub-Saharan Arica. The results therefore have implications for policy and practice in numerous similar contexts.

### Engendering gender-sensitive and equitable policies

The key implication of our study is that gender-sensitive and equitable policies and programs are essential for aligning community-based care with the social justice aims of primary health care and the SDGs. Gender-sensitive policies and programs ideally transform discriminatory gender norms progressively, and at the very least, do not perpetuate harmful gender stereotypes (Morgan *et al*., 2016). Expecting women to work in challenging conditions and for levels of remuneration that are deemed unacceptable by men (as they were in this study) and/or in conditions which they have no say in deciding (as the women caregivers in this study had no say) perpetuates gendered discrimination (Morgan *et al*., 2016; Steege *et al*., 2018). It relies on the pervasive belief that caring is women’s natural role, to justify what would otherwise be considered grossly inadequate conditions. To achieve the social justice aims of primary health care and the gender equality targets of the SDGs, our results concur with those of other research indicating that it is essential to ensure caregivers voices are included in decision making (Daniels *et al*., 2012).

### Moving beyond communities ‘helping themselves’

Our results further indicate that improving the material conditions in which community-based care is provided, is essential for achieving gender equality. Gendered norms and expectations are partly shaped by material conditions, including women’s economic dependence (Nussbaum, 2000). Implementing material interventions, like valuing the work women perform by remunerating them and/or assisting them with labour saving technology, could also contribute to attitudinal changes and the empowerment of women. Not having material resources and having no control over resource allocation, disempowered women caregivers in this community, as it has been shown to do in other CHW programs (Kane *et al*., 2016).

Lack of material resources also limited the effectiveness of community-based care for children. Given the high levels of poverty in this subsistence-farming community (Brear *et al*., 2018b) (and more generally in Swaziland and southern Africa), these results indicate that community-based responses will be unable to provide optimal (or even satisfactory) care for children affected by AIDS, in the absence of significant external material assistance. This is perhaps self-evident. However, the effect of extreme poverty that results in suboptimal levels of care and environments in which numerous barriers to health exist, receives insufficient attention. It is a feature of primary health care inspired models of community self-reliance that imply economically marginalised communities can become empowered in the absence of material resource redistribution (Campbell, 2014).

The challenging conditions in which predominantly female community-based caregivers perform their work are well recognised (Kane *et al*., 2016). However, these conditions are rarely conceptualised in policy debates as a gender issue (Daniels *et al*., 2012). Our results show that at the local-level community-based care work, and the conditions in which it was performed were shaped by discriminatory gender norms, specifically the belief that caregiving is ‘women’s work’. The gender dynamics are neglected in Swazi policies which allude to entire communities mobilising to provide care to children affected by AIDS. Notably, the extent to which these policies are informed by the voices and experiences of women who provide care is limited.

### Including women’s voices

Including women’s voices in research is a cornerstone of gender analysis (Morgan *et al*., 2016). Our results, which show stark differences between community-based care as experienced by women, as compared to how it is conceptualised in policy and programs, demonstrate the importance of this approach. They reveal that women were not even aware of policy recommendations and/or their own entitlements, and certainly played no role in deciding how resources were allocated, or whether caregiving was remunerated. They clearly wanted to receive stipends, a desire which is denigrated in Swazi policy documents which conceptualise remunerating care as undermining altruistic motivations for caregiving (UNICEF Swaziland, 2012).

## Conclusion

The study highlights the importance of hearing and legitimising the voices of marginalised women who provide community-based care, and contributing them to the research knowledge that informs primary health care policies and programs. The community voices that contributed to this research showed that the framework for community-based care of children affected by AIDS and the Swazi policies and programs based on it, perpetuate gender inequality. The reliance on women’s unpaid work enshrined in community-based care is incompatible with the social justice principles of primary health care and antithetical to numerous SDGs. Strategies to value care-related labour, including by providing caregivers remuneration and labour-saving technologies, would also contribute to aligning community-based care approaches with the social justice and developmental principles of international declarations. Increasing material assistance to communities and women caring for children affected by AIDS is also essential for ensuring children receive an adequate standard of care.
